# 172. Impact of a quality improvement re-training program on the blood culture volume, yield and contamination rates; experience from South India

**DOI:** 10.1093/ofid/ofad500.245

**Published:** 2023-11-27

**Authors:** Kaamil Zubair Rabbani Amaanulla, Rajalakshmi Ananthanarayanan, Karthik Asok Geetha, Rukhsar Abdur Rahim Mulla, Vettakkara Kandy Muhammed Niyas, Ashalatha Nair, Jeffrey Jomes, Febeena Hussain

**Affiliations:** KIMSHEALTH Trivandrum, Thiruvananthapuram, Kerala, India; Kerala Institute of Medical Sciences, Trivandrum, Kerala, India; KIMSHEALTH Trivandrum, Thiruvananthapuram, Kerala, India; KIMSHEALTH Trivandrum, Thiruvananthapuram, Kerala, India; KIMSHEALTH, Thiruvananthapuram, Trivandrum, Kerala, India; KIMSHEALTH Trivandrum, Thiruvananthapuram, Kerala, India; KIMSHEALTH Trivandrum, Thiruvananthapuram, Kerala, India; KIMSHEALTH Trivandrum, Thiruvananthapuram, Kerala, India

## Abstract

**Background:**

Blood cultures help to detect causative agents in bacteremia and are important for targeted therapy. Adequate blood culture volume is considered as 8-10ml in each bottle and at least 2 aerobic and 2 anaerobic blood culture bottles are recommended. Inadequate blood culture fills are common and may affect yield.

Figure 1

**Figure:**
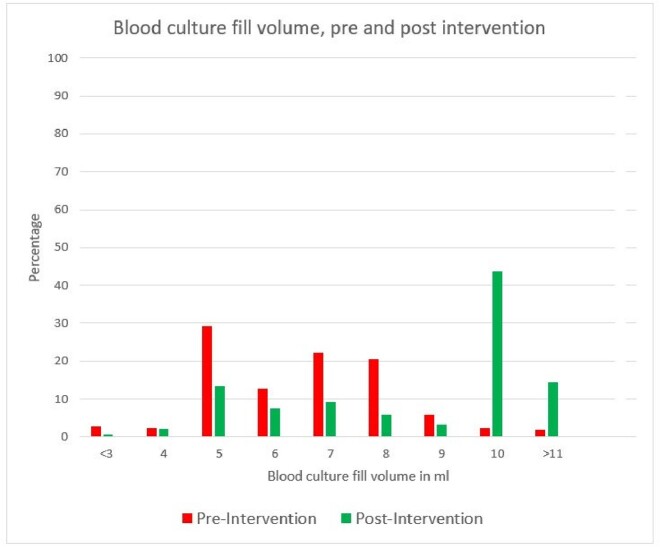

**Methods:**

The study was done in our healthcare facility-KIMSHEALTH, Trivandrum, India. Pre-intervention period was between May 2021 and June 2021; post-intervention was between May 2022 and June 2022. The intervention was through plan/do/study/act (PDSA) cycles and included multiple training sessions to the phlebotomy team regarding adequate fill volume which is 8-10 ml in each blood culture bottle. Training for maintaining asepsis during collection was reinforced. All blood culture samples received in the hospital microbiology laboratory during the above period were included in this study and compared between the pre-intervention and post-intervention period. Pediatric blood culture samples were excluded.

**Results:**

Blood culture samples studied during pre and post intervention period were 1428 and 1716 respectively. In the pre-intervention phase adequate fill volume was noted in 30.81% bottles (figure 1), while in the post intervention adequate fill volume was noted in 68.06% bottles *(p < 0.01)*. Blood culture contamination rate was 3.01% in the pre-intervention period which improved to 2% in the post-intervention period and the difference was significant *(p< 0.01).* Blood culture yield of a pathogenic organism did not show significant difference after the intervention (11.2% in pre-intervention group Vs 11.45% in post intervention group).

**Conclusion:**

In this study, an improvement was noted in the blood culture fills by 38% and contamination rate reduced by 1%. Though there was no significant increase in blood culture yield with intervention, the reduction in contamination was noted which will reduce unnecessary antibiotic initiation.

**Disclosures:**

**All Authors**: No reported disclosures

